# Entrustable professional activities: a roadmap for infectious diseases fellowship training in antibiotic stewardship

**DOI:** 10.1017/ash.2026.10767

**Published:** 2026-06-23

**Authors:** Reinaldo Perez, Molly McDonough, Eileen Maziarz, Rebekah H. Wrenn, Rebekah Moehring

**Affiliations:** 1 Duke University, Durham, NC, USA; 2 Durham VA Medical Center, Durham, NC, USA

## Abstract

Infectious diseases fellowship programs for physicians seek to train the next generation of leaders in antimicrobial stewardship, but few published resources are available to guide educational experiences. As an adaptable tool for training programs, we created a list of entrustable professional activities and suggested tasks to achieve competency during fellowship.

## Introduction

Guidelines published by the Society for Healthcare Epidemiology of America (SHEA) and Infectious Diseases Society of America (IDSA) have long advocated for infectious disease (ID) physician and pharmacist co-leadership in antibiotic stewardship programs.^1^ Recognizing a gap in resources, the IDSA launched a core curriculum for antibiotic stewardship for use by clinical ID fellowship programs.^2^ The course has been a success with over 159 programs adapting this material, high learner satisfaction, and the impending publication of an advanced antimicrobial stewardship curriculum.^3^ However, subsequent qualitative work evaluating ID fellow learning preferences has suggested this foundation is insufficient in preparing trainees for stewardship practice and program leadership. The study identified a strong preference for “skills, not just knowledge” and opportunities for experiential learning in the day-to-day work of antimicrobial stewardship.^4^

The Duke Center for Antimicrobial Stewardship and Infection Prevention (DCASIP) seeks to provide a robust, hands-on training experience for physician ID fellows. Training needs differ with certain fellows seeking basic competencies for ID clinical practice, while others aspire to a career in antimicrobial stewardship program leadership. To meet the needs of our ID fellows, we developed a list of core stewardship entrustable professional activities (EPAs) and tasks to facilitate competency, allowing differentiation for basic and advanced training.

EPAs are a commonly used tool in competency-based models of health professions education. EPAs are, “units of clinical practice that, as a whole, can be entrusted to a professional, or someone in training for that profession.” Assessment of competency attainment for EPAs can be performed using retrospective and prospective entrustment scales which can inform programmatic evaluations of readiness for independent clinical practice.^5^ We sought to create a tool to aid in the instruction and assessment of physician fellows at our institution and serve as a template for other ID physician training programs.

## Methods

Antimicrobial stewardship core EPAs were identified based on published 2014 SHEA guidance for knowledge and skills required for antimicrobial stewardship leaders, CDC’s core elements of antimicrobial stewardship, and interviews with DCASIP faculty and fellowship program leadership.^6,7^ We then identified specific tasks to be performed or resources to be discussed with mentors to facilitate competency attainment. EPAs were separated into skills we felt essential for all fellows in training (basic EPAs) and skills specific to trainees interested in stewardship program leadership as a career (advanced EPAs). Final stewardship EPAs were then mapped to Accreditation Council for Graduate Medical Education (ACGME) IDs milestones by consensus from the author group.

## Results

We identified 35 sets of activities (20 basic and 15 advanced) to facilitate competency attainment of EPAs needed for independent antibiotic stewardship practice. EPAs were organized around the CDC core elements of antimicrobial stewardship and key interdisciplinary partnerships. The information was organized into an easy-to-use table which we have used to track fellow development at ID fellow mentorship committee meetings. We adapted and generalized these activities to serve as a template document for other institutions to personalize with experiences at their own facilities. The final product is presented in Table 1 and Table 2.

**Table 1.** Entrustable professional activities for antimicrobial stewardship practice, by CDC’s antimicrobial stewardship core elements Table 1 long description.

Entrustable professional activity (EPA) by core elements	Tasks	Skill level	ACGME milestone
Hospital leadership commitment and accountability			
Understand roles and responsibilities of antimicrobial stewardship team members including physicians, pharmacists, infection preventionists, microbiologists, and hospital administrators	Attend ASP working meetings and relevant hospital committee meetings (eg, Pharmacy and Therapeutics committee) Discuss career path and training with physician and pharmacist ASP leaders Review institutional ASP policy	Basic	MK 4 SBP 5 ICS 2 ICS 3
Describe the interpersonal skills and interprofessional needs to sustain an ASP	Participate in routine prospective audit and feedback Participate in handshake stewardship rounds Shadow an ID pharmacist	Basic	PC 3 MK 4 SBP 2 SBP 4 PROF 1 ICS 2
Compare and contrast how to obtain clinician and administrative buy in for a program or intervention	Participate in a needs assessment for a potential stewardship intervention	Basic	SBP 2 ICS 2 ICS 3
Describe IT resources available at an institution for performing antimicrobial stewardship activities	Meet with your division/department’s EMR superuser or IT lead to better understand the resources available	Basic	SBP 5
Write and present a strategic plan and business case to institutional leadership for stewardship resources	Participate in or review a business proposal prepared by your facility’s ASP	Advanced	SBP 1 SBP 2 SBP 5 ICS 3
Develop prioritized approaches to implementing stewardship activities when resources are limited	Meet and discuss resource limitations and models for stewardship activities with leads at a smaller community hospital, public health department, or outlying clinic (Where available) Attend system- or network-level stewardship working meeting	Advanced	MK 4 SBP 4 SBP 5 ICS 2 ICS 3
Pharmacy expertise			
Assess and respond to antimicrobial or microbiology lab related shortages	Aid in creating a guidance document for an impending shortage Review the American Society of Health-System Pharmacists (ASHP) shortage website	Basic	MK 3 MK 4 SBP 3 SBP 4 ICS 3
Understand common mechanisms of resistance for different antimicrobial/organism combinations and their impact on resistance to other antimicrobials	Review the genotypic and phenotypic resistance testing available through the local microbiology lab	Basic	MK 3 MK 4 MK 5
Assess a hospital’s antimicrobial formulary including stewardship strategy (eg, restriction, monitoring parameters), use cases, cost and efficacy	Partner with a pharmacist to participate in preparation of a medication use evaluation (MUE) Partner with a pharmacist to update your facility’s current restricted antimicrobial list	Advanced	MK 3 MK 4 SBP 3 ICS 2
Understand antimicrobial therapy options for highly resistant organisms	Review IDSA Resistant Gram Negative Guidance	Basic	MK 3 PBLI 1
	Participate in review of your facility’s epidemiology data for drug-resistant pathogens	Advanced	MK 3 SBP 4 SBP 5 PBLI 1
Action			
Summarize the pros and cons of restriction of antimicrobial therapy and postprescription prospective audit and feedback	Review the SHEA/IDSA guidelines for implementation of an antibiotic stewardship program Review/approve requests for restricted antibiotics Participate in routine prospective audit and feedback	Basic	MK 4 SBP 5 PBLI 1 ICS 2
	Participate in ASP review and update of your facility’s restricted antimicrobial list	Advanced	SBP 2 ICS 3
Develop institutional guidelines for antimicrobial use	Assist your ASP in creating a new treatment guideline or in updating an existing one	Basic	MK 4 PBLI 1 ICS 3
Evaluate approaches for IV to PO conversion	Review your hospital’s IV to PO conversion policy	Advanced	SBP1 SBP 3
Describe types of allergic reactions to antimicrobials and methods to optimize allergy assessment	Review the trial data for direct oral penicillin challenge Review cephalosporin cross-reactivity charts Understand facility policy or standard operating procedure for allergy assessment	Basic	MK 3 MK 4 PBLI 1
Compare and contrast associations between use of specific antimicrobial agents and development of resistance or *C. difficile*	Review SHEA compendium on strategies to reduce hospital onset *C. difficile* infection (HO-CDI) Participate in case reviews of HO-CDI events, with emphasis on antibiotic use appropriateness Review IDSA guidelines for resistant gram-negative infections and management of *C. difficile*	Basic	MK 4 PBLI 1 SBP 1 SBP 2
Apply national guidelines for infectious diseases to institutional guidance relevant to antimicrobial stewardship	Review IDSA guidelines for intra-abdominal infections, diabetic foot infections, pneumonia, asymptomatic bacteriuria, neutropenic fever, vertebral osteomyelitis and skin/soft tissue infections Compare national guidelines to your facility’s institutional guideline, incorporating the context of your facility’s patient populations, local epidemiologic data (eg, antibiogram), and formulary	Advanced	MK 3 MK 4 PBLI 1 PBLI 2
Tracking and reporting			
Understand methods, data needs, and interpretation of antimicrobial use metrics Compare standard methods of measuring antimicrobial use, including metrics such as DDD, DOT, and LOT	Read a review of antibiotic use metrics Participate in your ASP’s routine review of facility AU data and reporting	Basic	MK4 SBP 2
	Evaluate facility or unit performance based on AU metrics and identify opportunities for intervention Present AU metrics to a unit’s leadership with opportunities for action	Advanced	MK 4 MK 6 SBP 2
Recognize approaches to benchmarking antimicrobial use within and across institutions Understand processes and definitions associated with the NHSN AUR module	Read the NHSN AUR Option Protocol Review reports available for your facility through the NHSN AUR module	Basic	MK 4 SBP 1 SBP 2 SBP 5
Discuss approaches to measure the impact of a stewardship program or stewardship intervention and outcomes relevant to hospital leadership	Meet with your facility’s ASP co-leads and review the annual metrics they present to hospital leadership to justify program costs Review the joint commission requirements for antibiotic stewardship programs	Advanced	MK 4 SBP 1 SBP 2 SBP 5
Describe the unique data challenges in AU data in outpatient vs inpatient setting	Evaluate a published stewardship intervention in the inpatient and outpatient setting and discuss at a journal club	Advanced	MK 4 SBP 1 SBP 2 SBP 5 PBLI 1
Education			
Explain the role and limitations of education regarding appropriate antimicrobial use Describe the wide array of hospital staff involved in antimicrobial ordering and administration Generate educational materials appropriate to different levels of learners including clinicians, nurses, patients/caregivers, and the wider public	Participate in the development or revision of an institutional guideline for management of an infection Present on stewardship topic to a relevant group at your facility (resident conference, physician group, nurses, pharmacists, or other opportunity)	Basic	MK 4 ICS 2 ICS 3 SBP 2 SBP 5

*Note:* Full ACGME milestone definitions and bibliography for referenced educational materials are available in the supplement.

ACGME, Accreditation Council for Graduate Medical Education; ASP, antibiotic stewardship program; ID, infectious diseases; IT, information technology; EMR, electronic medical record; NHSN, National Healthcare Safety Network; AUR, antibiotic use and resistance; DDD, defined daily doses; SOT, days of therapy; LOT, length of therapy; AU, antimicrobial use; PC, patient care; MK, medical knowledge; PBLI, practice-based learning and improvement; SBP, systems-based practice; PROF, Professionalism; ICS, interpersonal and communication skills.

**Table 2.** Entrustable professional activities for antimicrobial stewardship partnerships and methodologic skills

Entrustable professional activity	Tasks	Skill level	ACGME milestone
Microbiology laboratory partnership			
Understand breakpoints for antimicrobial susceptibility testing and current stage of implementation locally	Review the CLSI M100 for common organism breakpoints and intrinsic resistance patterns Discuss with your microbiology lab which platform they are using for susceptibility testing and its limitations Review the lab’s “breakpoints in use” document used for College of American Pathologists (CAP) accreditation	Basic	MK 2 MK 3
Evaluate opportunities for diagnostic stewardship within the lab and understand the interventions currently in place at your institution	Participate in meeting with your ASP and/or microbiology lab leadership to discuss current diagnostic stewardship projects and strategy	Basic	MK 1 MK 2 PBLI 1 SBP 2
Understand CLSI recommendations for constructing institutional antibiograms	Assist in creating or reviewing an antibiogram for your facility	Basic	MK 2 MK 4
Summarize the importance and effects of a cascaded or tiered reporting strategy Describe approaches to testing and reporting antimicrobial susceptibilities to promote antimicrobial stewardship principles Understand effects of laboratory standard procedures that affect reporting to clinicians	Review current CLSI guidance for tiered reporting of antimicrobial susceptibility testing (M100) Review local cascade reporting rules for specific pathogens Review facility’s lab SOPs for reporting of sputum cultures and other cultures from non-sterile sites	Advanced	MK 2 MK 3 MK 4 SBP 2
Understand the pros and cons of using an antibiogram to assist with decisions on recommendations for empirical therapy	Assist in updating institutional empiric treatment guidance after the annual antibiogram update	Advanced	MK 2 MK 4 SBP 2 SBP 4 SBP 5 ICS 3
Infection prevention partnership			
Discuss basic infection prevention principles and processes and the interrelationship with ASP	Attend an infection prevention workgroup meeting at your facility Shadow an infection preventionist	Basic	MK 4 SBP 1 SBP 2
Explain the differences between surveillance definitions and clinical definitions for healthcare associated infections	Review NHSN classifications for CLABSI, CAUTI, HO-CDI and SSI	Basic	SBP 2 MK 4
Understand current recommendations for use of antibiotics for surgical prophylaxis	Review your facility’s perioperative prophylaxis guideline Meet with or shadow perioperative staff to understand process for ordering and administering perioperative antibiotic use	Basic	PBLI 1 SBP 2
Quality improvement principles and practice			
Use quality improvement methodologies including root cause analysis and PDSA cycles to improve antimicrobial use.	Participate in a root cause analysis. Apply PDSA cycles for at least one stewardship intervention Summarize/Present QI Implementation project at a local committee meeting, or local conference	Advanced	MK 4 MK 6 SBP 1 SBP 2
Compare and contrast the application of quality improvement and implementation science methods for application in ASP PDSA, Lean, Six Sigma Implementation science Human factor design Organizational change Failure modes and effect analysis Root cause analysis	Review educational material related to advanced techniques in quality improvement Participate in an ongoing or new quality improvement project in which one or more quality improvement techniques are applied Review SHEA White paper on implementation science	Advanced	SBP 1 SBP 2
Understand the analytic methods commonly used in AS research	Review the SHEA Research Methods white papers, particularly for randomized controlled trials, mixed methods studies, observational studies, and quasi-experimental studies Apply principles described in any of the SHEA Research Methods white papers to a research or QI project Summarize/Present QI Implementation project at Antimicrobial Stewardship committee, a local quality conference, or a national meeting	Advanced	MK 4 MK 6 SBP 2

*Note:* Full ACGME milestone definitions and referenced educational materials are available in the supplement.

ACGME, Accreditation Council for Graduate Medical Education; ASP, antibiotic stewardship program; CLSI, Clinical Laboratory Standards Institute; FDA, Food and Drug Administration; SOP, standard operating procedure; NHSN, National Healthcare Safety Network; CLABSI, central line-associated bloodstream infection; CAUTI, catheter-associated UTI; HO-CDI, hospital-onset Clostridioides difficile infection; SSI, surgical site infection; AUR, antibiotic use and resistance; PDSA, Plan Do Study Act; QI, quality improvement; PC, patient care; MK, medical knowledge; PBLI, practice-based learning and improvement; SBP, systems-based practice; PROF, Professionalism; ICS, interpersonal and communication skills.

## Discussion

In the 12 years since the publication of the original SHEA white paper outlining skills for antibiotic stewards, our field has continued to grow and evolve.^6^ Importantly, dedicated training in antibiotic stewardship is increasingly incorporated into clinical ID fellowship training with the ACGME including a core requirement that fellows demonstrate “sufficient knowledge in antimicrobial stewardship.”^8,9^ As a tool for IDs fellowship programs, we created this framework of individual EPAs and suggested tasks to achieve them. Further, we suggest activities for fellows aiming to achieve basic competencies, differentiated from those suggested for advanced training to prepare fellows for careers in antimicrobial stewardship program leadership.

Previously published resources for training antimicrobial stewards have largely focused on curricula and ready-to-use educational materials such as lectures, case discussions, or role-playing exercises.^2^ Although these are essential tools, they focus on introductory learners and foundational knowledge. We sought to build on this foundation and create a tool that could be used by faculty and program leadership for ongoing mentorship and assessment of trainees’ development within established ACGME frameworks.^9^ Although the use of EPAs is well described for medicine residents, we could find no literature on use of EPAs for ID fellowship training and feel they could be a powerful tool to enhance trainee evaluation. Prior qualitative research on stewardship curricula identified a desire for experiential learning, interaction with multidisciplinary teams, and an emphasis on understanding programmatic features beyond antibiotic restriction and prospective audit and feedback.^4^ We address this by providing fellows and faculty with discrete opportunities available at our program to develop these programmatic leadership skills. Importantly, these specific tasks allow ID fellows to work directly and learn from ID pharmacist champions, understanding the importance of co-leadership in implementation of antimicrobial stewardship. Further, these EPAs emphasize key clinical partnerships with microbiology and infection prevention teams and suggest specific skills in quality improvement and epidemiologic methods that have high value in assessing program needs and strategy.

Trainees may be considering employment where they will be expected to participate in stewardship activities or co-lead a program without clear knowledge of the experiences needed to lead a program successfully. Importantly our tool includes the development of “basic” and “advanced” pathways allowing the training program to adapt to individual needs. Though Duke is still early in the implementation of this tool, our fellows, faculty, and mentorship committees have found it advantageous in providing structure and identifying opportunities for growth. We hope that in sharing resources, we can collaborate with other institutions in preparing the next generation of leaders in antimicrobial stewardship.

These proposed EPAs have limitations. The tasks are based on experiences we have been able to offer trainees at a large academic tertiary care center or by participation in a large, collaborative stewardship network with robust data resources.^10^ Thus, not all the suggested experiences may be feasible at other programs. We encourage readers to use this table as a template and then adjust activities that support competency attainment based on those available in their programs. The EPAs are based on the published literature and the expert opinion of our single-center expertise and are not intended to be all-encompassing. Currently, we do not have formal evaluative data of the proposed EPAs or their reception by trainees or faculty outside of our institution. Due to our small sample size of four fellows per year, we felt collection of such data would be infeasible in a timely manner and thus favored the benefit of sharing a potentially useful tool now. Despite these limitations, we feel the proposed EPAs and tasks could provide an example framework for local adaptation for fellowship programs seeking to provide greater structure for a differentiated approach to stewardship training during ID fellowship.

Antibiotic stewardship programs continue to grow outside of the traditional inpatient setting, serving outpatient clinics, nursing homes, and working within multiple layers of administration across increasingly large healthcare systems. Preparing our trainees to be successful in these diverse arenas will require thoughtful mentorship and curated experiences. Intentional tools such as ours and increased collaboration across centers to facilitate these experiences will be essential to meeting the challenge of antimicrobial resistance.

## Supporting information

10.1017/ash.2026.10767.sm001 Perez et al. supplementary material Perez et al. supplementary material

## Supplementary material

The supplementary material for this article can be found at https://doi.org/10.1017/ash.2026.10767.

## Table 1. Long description

The table presents 35 sets of activities categorized into basic and advanced levels to facilitate competency attainment for independent antibiotic stewardship practice. It is organized around the CDC core elements of antimicrobial stewardship and key interdisciplinary partnerships. The table includes columns for tasks, skill level, and ACGME milestones, with rows detailing specific activities and their corresponding skill levels and milestones. The table is divided into sections such as Hospital leadership commitment and accountability, Pharmacy expertise, Action, Tracking and reporting, and Education. Each section lists specific tasks and the required skill levels and milestones for each task. The table is used to track fellow development at ID fellow mentorship committee meetings and serves as a template for other institutions to personalize with their own experiences.

Navigate back to Table 1..
