# Supplementary material for: Entrustable professional activities: a roadmap for infectious diseases fellowship training in antibiotic stewardship
Source: Antimicrob Steward Healthc Epidemiol. 2026 Jun 23;6(1):e188. doi: 10.1017/ash.2026.10767 (PMC13312247; doi:10.1017/ash.2026.10767)
Supplement: Perez et al. supplementary material [file S2732494X26107670sup001.docx]

**Tables.**

Table 1. Entrustable Professional Activities for Antimicrobial Stewardship Practice, by CDC’s Antimicrobial Stewardship Core Elements

| **Entrustable Professional Activity (EPA) by Core Elements** | **Tasks** | **Skill Level** | **ACGME Milestone**[1] |
| --- | --- | --- | --- |
| **Hospital Leadership Commitment and Accountability** |  |  |  |
| Understand roles and responsibilities of antimicrobial stewardship team members including physicians, pharmacists, infection preventionists, microbiologists, and hospital administrators | Attend ASP working meetings and relevant hospital committee meetings (e.g. Pharmacy and Therapeutics committee)  Discuss career path and training with physician and pharmacist ASP leaders  Review institutional ASP policy | Basic | MK 4  SBP 5  ICS 2  ICS 3 |
| Describe the interpersonal skills and interprofessional needs to sustain an ASP | Participate in routine prospective audit and feedback  Participate in handshake stewardship rounds  Shadow an ID pharmacist | Basic | PC 3  MK 4  SBP 2  SBP 4  PROF 1  ICS 2 |
| Compare and contrast how to obtain clinician and administrative buy in for a program or intervention | Participate in a needs assessment for a potential stewardship intervention | Basic | SBP 2  ICS 2  ICS 3 |
| Describe IT resources available at an institution for performing antimicrobial stewardship activities | Meet with your division/department’s EMR superuser or IT lead to better understand the resources available | Basic | SBP 5 |
| Write and present a strategic plan and business case to institutional leadership for stewardship resources | Participate in or review a business proposal prepared by your facility’s ASP | Advanced | SBP 1  SBP 2  SBP 5  ICS 3 |
| Develop prioritized approaches to implementing stewardship activities when resources are limited | Meet and discuss resource limitations and models for stewardship activities with leads at a smaller community hospital, public health department, or outlying clinic  (Where available) Attend system- or network-level stewardship working meeting | Advanced | MK 4  SBP 4  SBP 5  ICS 2  ICS 3 |
| **Pharmacy Expertise** |  |  |  |
| Assess and respond to antimicrobial or microbiology lab related shortages | Aid in creating a guidance document for an impending shortage  Review the American Society of Health-System Pharmacists (ASHP) shortage website[2] (<https://www.ashp.org/drug-shortages>) | Basic | MK 3  MK 4  SBP 3  SBP 4  ICS 3 |
| Understand common mechanisms of resistance for different antimicrobial/organism combinations and their impact on resistance to other antimicrobials | Review the genotypic and phenotypic resistance testing available through the local microbiology lab | Basic | MK 3  MK 4  MK 5 |
| Assess a hospital’s antimicrobial formulary including stewardship strategy (e.g. restriction, monitoring parameters), use cases, cost and efficacy | Partner with a pharmacist to participate in preparation of a medication use evaluation (MUE)  Partner with a pharmacist to update your facility’s current restricted antimicrobial list | Advanced | MK 3  MK 4  SBP 3  ICS 2 |
| Understand antimicrobial therapy options for highly resistant organisms | Review IDSA Resistant Gram Negative Guidance[3] | Basic | MK 3  PBLI 1 |
|  | Participate in review of your facility’s epidemiology data for drug-resistant pathogens | Advanced | MK 3  SBP 4  SBP 5  PBLI 1 |
| **Action** |  |  |  |
| Summarize the pros and cons of restriction of antimicrobial therapy and post-prescription prospective audit and feedback | Review the SHEA/IDSA guidelines for implementation of an antibiotic stewardship program[4]  Review/approve requests for restricted antibiotics  Participate in routine prospective audit and feedback | Basic | MK 4  SBP 5  PBLI 1  ICS 2 |
|  | Participate in ASP review and update of your facility’s restricted antimicrobial list | Advanced | SBP 2  ICS 3 |
| Develop institutional guidelines for antimicrobial use | Assist your ASP in creating a new treatment guideline or in updating an existing one | Basic | MK 4  PBLI 1  ICS 3 |
| Evaluate approaches for IV to PO conversion | Review your hospital’s IV to PO conversion policy | Advanced | SBP1  SBP 3 |
| Describe types of allergic reactions to antimicrobials and methods to optimize allergy assessment | Review the trial data for direct oral penicillin challenge[5]  Review cephalosporin cross reactivity charts[6]  Understand facility policy or standard operating procedure for allergy assessment | Basic | MK 3  MK 4  PBLI 1 |
| Compare and contrast associations between use of specific antimicrobial agents and development of resistance or *C. difficile* | Review SHEA compendium on strategies to reduce hospital onset *C. difficile* infection (HO-CDI)[7]  Participate in case reviews of HO-CDI events, with emphasis on antibiotic use appropriateness  Review IDSA guidelines for resistant gram-negative infections and management of *C. difficile* | Basic | MK 4  PBLI 1  SBP 1  SBP 2 |
| Apply national guidelines for infectious diseases to institutional guidance relevant to antimicrobial stewardship | Review IDSA guidelines for intra-abdominal infections, diabetic foot infections, pneumonia, asymptomatic bacteriuria, neutropenic fever, vertebral osteomyelitis and skin/soft tissue infections[3,8–16]  Compare national guidelines to your facility’s institutional guideline, incorporating the context of your facility’s patient populations, local epidemiologic data (e.g. antibiogram), and formulary | Advanced | MK 3  MK 4  PBLI 1  PBLI 2 |
| **Tracking and Reporting** |  |  |  |
| Understand methods, data needs, and interpretation of antimicrobial use metrics  Compare standard methods of measuring antimicrobial use, including metrics such as DDD, DOT, and LOT | Read a review of antibiotic use metrics[17]  Participate in your ASP’s routine review of facility AU data and reporting | Basic | MK4  SBP 2 |
|  | Evaluate facility or unit performance based on AU metrics and identify opportunities for intervention  Present AU metrics to a unit’s leadership with opportunities for action | Advanced | MK 4  MK 6  SBP 2 |
| Recognize approaches to benchmarking antimicrobial use within and across institutions  Understand processes and definitions associated with the NHSN AUR module | Read the NHSN AUR Option Protocol[18]  Review reports available for your facility through the NHSN AUR module | Basic | MK 4  SBP 1  SBP 2  SBP 5 |
| Discuss approaches to measure the impact of a stewardship program or stewardship intervention and outcomes relevant to hospital leadership | Meet with your facility’s ASP co-leads and review the annual metrics they present to hospital leadership to justify program costs  Review the joint commission requirements for antibiotic stewardship programs[19] | Advanced | MK 4  SBP 1  SBP 2  SBP 5 |
| Describe the unique data challenges in AU data in outpatient vs inpatient setting | Evaluate a published stewardship intervention in the inpatient and outpatient setting and discuss at a journal club | Advanced | MK 4  SBP 1  SBP 2  SBP 5  PBLI 1 |
| **Education** |  |  |  |
| Explain the role and limitations of education regarding appropriate antimicrobial use  Describe the wide array of hospital staff involved in antimicrobial ordering and administration  Generate educational materials appropriate to different levels of learners including clinicians, nurses, patients/caregivers, and the wider public | Participate in the development or revision of an institutional guideline for management of an infection  Present on stewardship topic to a relevant group at your facility (resident conference, physician group, nurses, pharmacists, or other opportunity) | Basic | MK 4  ICS 2  ICS 3  SBP 2  SBP 5 |

**ACGME= Accreditation Council for Graduate Medical Education; ASP = Antibiotic Stewardship Program; ID= Infectious diseases; IT = information Technology; EMR = electronic medical record; NHSN = National healthcare safety setwork; AUR = Antibiotic use and resistance; DDD = defined daily doses; SOT = days of therapy; LOT = length of therapy; AU = antimicrobial use; PC = patient care; MK = medical knowledge; PBLI = Practice based learning and improvement; SBP = systems-based practice; PROF = Professionalism; ICS = Interpersonal and Communication Skills.**

**Table 2:** Entrustable Professional Activities **for Antimicrobial Stewardship Partnerships and Methodologic Skills**

| **Entrustable Professional Activity** | **Tasks** | **Skill Level** | **ACGME Milestone** |
| --- | --- | --- | --- |
| **Microbiology Laboratory Partnership** |  |  |  |
| Understand breakpoints for antimicrobial susceptibility testing and current stage of implementation locally | Review the CLSI M100 for common organism breakpoints and intrinsic resistance patterns[20]  Discuss with your microbiology lab which platform they are using for susceptibility testing and its limitations  Review the lab’s “breakpoints in use” document used for College of American Pathologists (CAP) accreditation[21] | Basic | MK 2  MK 3 |
| Evaluate opportunities for diagnostic stewardship within the lab and understand the interventions currently in place at your institution | Participate in meeting with your ASP and/or microbiology lab leadership to discuss current diagnostic stewardship projects and strategy | Basic | MK 1  MK 2  PBLI 1  SBP 2 |
| Understand CLSI recommendations for constructing institutional  antibiograms | Assist in creating or reviewing an antibiogram for your facility | Basic | MK 2  MK 4 |
| Summarize the importance and effects of a cascaded or tiered reporting strategy  Describe approaches to testing and reporting antimicrobial susceptibilities to promote antimicrobial stewardship principles  Understand effects of laboratory standard procedures that affect reporting to clinicians | Review current CLSI guidance for tiered reporting of antimicrobial susceptibility testing (M100)[20]  Review local cascade reporting rules for specific pathogens  Review facility’s lab SOPs for reporting of sputum cultures and other cultures from non-sterile sites | Advanced | MK 2  MK 3  MK 4  SBP 2 |
| Understand the pros and cons of using an antibiogram to assist with decisions on recommendations for empirical therapy | Assist in updating institutional empiric treatment guidance after the annual antibiogram update | Advanced | MK 2  MK 4  SBP 2  SBP 4  SBP 5  ICS 3 |
| **Infection Prevention Partnership** |  |  |  |
| Discuss basic infection prevention principles and processes and the interrelationship  with ASP | Attend an infection prevention workgroup meeting at your facility    Shadow an infection preventionist | Basic | MK 4  SBP 1  SBP 2 |
| Explain the differences between surveillance definitions and clinical  definitions for healthcare associated infections | Review NHSN classifications for CLABSI, CAUTI, HO-CDI and SSI[22] | Basic | SBP 2  MK 4 |
| Understand current recommendations for use of antibiotics for surgical prophylaxis | Review your facility’s perioperative prophylaxis guideline  Meet with or shadow perioperative staff to understand process for ordering and administering perioperative antibiotic use | Basic | PBLI 1  SBP 2 |
| **Quality Improvement Principles and Practice** |  |  |  |
| Use quality improvement methodologies including root cause analysis and PDSA cycles to improve antimicrobial use. | Participate in a root cause analysis.  Apply PDSA cycles for at least one stewardship intervention  Summarize/Present QI Implementation project at a local committee meeting, or local conference | Advanced | MK 4  MK 6  SBP 1  SBP 2 |
| Compare and contrast the application of quality improvement and implementation science methods for application in ASP   - PDSA, Lean, Six Sigma - Implementation science - Human factor design - Organizational change - Failure modes and effect analysis - Root cause analysis | Review educational material related to advanced techniques in quality improvement[23]  Participate in an ongoing or new quality improvement project in which one or more quality improvement techniques are applied  Review SHEA White paper on implementation science[24] | Advanced | SBP 1  SBP 2 |
| Understand the analytic methods commonly used in AS research | Review the SHEA Research Methods white papers, particularly for randomized controlled trials, mixed methods studies, observational studies, and quasi-experimental studies[25]  Apply principles described in any of the SHEA Research Methods white papers to a research or QI project  Summarize/Present QI Implementation project at Antimicrobial Stewardship committee, a local quality conference, or a national meeting | Advanced | MK 4  MK 6  SBP 2 |

**ACGME= Accreditation Council for Graduate Medical Education; CLSI = Clinical Laboratory Standards Institute; FDA = Food and Drug Administration; ASP = Antibiotic Stewardship Program; SOP = standard operating procedure; NHSN = National healthcare safety setwork; CLABSI = central line associated bloodstream infection; CAUTI = catheter associated UTI; HO-CDI = hospital onset Clostridioides difficile infection; SSI = surgical site infection; AUR = Antibiotic use and resistance; PDSA = Plan Do Study Act; ASP = Antibiotic Stewardship Program; QI = Quality Improvement; PC = patient care; MK = medical knowledge; PBLI = Practice based learning and improvement; SBP = systems-based practice; PROF = Professionalism; ICS = Interpersonal and Communication Skills.**

1. Accreditation Council for Graduate Medical Education. Infectious Disease Milestones. 2021; Available at: https://www.acgme.org/globalassets/PDFs/Milestones/InfectiousDiseaseMilestones2.0.pdf?ver=2021-04-22-144052-870.

2. Current Drug Shortages - ASHP. Available at: https://www.ashp.org/Drug-Shortages/Current-Shortages. Accessed 7 April 2026.

3. Infectious Diseases Society of America 2024 Guidance on the Treatment of Antimicrobial-Resistant Gram-Negative Infections | Clinical Infectious Diseases | Oxford Academic. Available at: https://academic.oup.com/cid/advance-article/doi/10.1093/cid/ciae403/7728556?login=true. Accessed 7 April 2026.

4. Barlam TF, Cosgrove SE, Abbo LM, et al. Implementing an Antibiotic Stewardship Program: Guidelines by the Infectious Diseases Society of America and the Society for Healthcare Epidemiology of America. Clin Infect Dis **2016**; 62:e51–e77.

5. Copaescu AM, Vogrin S, James F, et al. Efficacy of a Clinical Decision Rule to Enable Direct Oral Challenge in Patients With Low-Risk Penicillin Allergy: The PALACE Randomized Clinical Trial. JAMA Intern Med **2023**; 183:944–952.

6. D’Errico S, Frati P, Zanon M, et al. Cephalosporins’ Cross-Reactivity and the High Degree of Required Knowledge. Case Report and Review of the Literature. Antibiotics **2020**; 9:209.

7. Strategies to prevent Clostridioides difficile infections in acute-care hospitals: 2022 Update | Infection Control & Hospital Epidemiology | Cambridge Core. Available at: https://www.cambridge.org/core/journals/infection-control-and-hospital-epidemiology/article/strategies-to-prevent-clostridioides-difficile-infections-in-acutecare-hospitals-2022-update/575A2A0C9E68BD8535D14B2E337FD0A4. Accessed 7 April 2026.

8. Practice Guidelines for the Diagnosis and Management of Skin and Soft Tissue Infections: 2014 Update by the Infectious Diseases Society of America | Clinical Infectious Diseases | Oxford Academic. Available at: https://academic.oup.com/cid/article/59/2/e10/2895845?login=true. Accessed 7 April 2026.

9. Senneville É, Albalawi Z, van Asten SA, et al. IWGDF/IDSA Guidelines on the Diagnosis and Treatment of Diabetes-related Foot Infections (IWGDF/IDSA 2023). Clin Infect Dis **2023**; :ciad527.

10. Johnson S, Lavergne V, Skinner AM, et al. Clinical Practice Guideline by the Infectious Diseases Society of America (IDSA) and Society for Healthcare Epidemiology of America (SHEA): 2021 Focused Update Guidelines on Management of Clostridioides difficile Infection in Adults. Clinical Infectious Diseases **2021**; 73:e1029–e1044.

11. Bonomo RA, Chow AW, Edwards MS, et al. 2024 Clinical Practice Guideline Update by the Infectious Diseases Society of America on Complicated Intra-abdominal Infections: Risk Assessment, Diagnostic Imaging, and Microbiological Evaluation in Adults, Children, and Pregnant People. Clin Infect Dis **2024**; 79:S81–S87.

12. America</p> <p>Joshua PM Grant W Waterer, Ann C Long, Antonio Anzueto, Jan Brozek, Kristina Crothers, Laura A Cooley, Nathan C Dean, Michael J Fine, Scott A Flanders, Marie R Griffin, Mark L Metersky, Daniel M Musher, Marcos I Restrepo, and Cynthia G Whitney; on behalf of the American Thoracic Society and Infectious Diseases Society of. Diagnosis and Treatment of Adults with Community-acquired Pneumonia. An Official Clinical Practice Guideline of the American Thoracic Society and Infectious Diseases Society of America. Available at: https://www.idsociety.org/practice-guideline/community-acquired-pneumonia-cap-in-adults/. Accessed 14 February 2023.

13. Nicolle LE, Gupta K, Bradley SF, et al. Clinical Practice Guideline for the Management of Asymptomatic Bacteriuria: 2019 Update by the Infectious Diseases Society of Americaa. Clinical Infectious Diseases **2019**; 68:e83–e110.

14. Metlay JP, Waterer GW, Long AC, et al. Diagnosis and Treatment of Adults with Community-acquired Pneumonia. An Official Clinical Practice Guideline of the American Thoracic Society and Infectious Diseases Society of America. Am J Respir Crit Care Med **2019**; 200:e45–e67.

15. Taplitz RA, Kennedy EB, Bow EJ, et al. Outpatient Management of Fever and Neutropenia in Adults Treated for Malignancy: American Society of Clinical Oncology and Infectious Diseases Society of America Clinical Practice Guideline Update. J Clin Oncol **2018**; 36:1443–1453.

16. Berbari EF, Kanj SS, Kowalski TJ, et al. 2015 Infectious Diseases Society of America (IDSA) Clinical Practice Guidelines for the Diagnosis and Treatment of Native Vertebral Osteomyelitis in Adultsa. Available at: https://dx.doi.org/10.1093/cid/civ482. Accessed 7 April 2026.

17. Yarrington ME, Moehring RW. Basic, Advanced, and Novel Metrics to Guide Antibiotic Use Assessments. Curr Treat Options Infect Dis **2019**; 11:145–160.

18. AUR | PSC | NHSN | CDC. 2026. Available at: https://www.cdc.gov/nhsn/psc/aur/index.html. Accessed 7 April 2026.

19. R3 Report Issue 35: New and Revised Requirements for Antibiotic Stewardship | Joint Commission. Available at: https://www.jointcommission.org/en-us/standards/r3-report/r3-report-35. Accessed 7 April 2026.

20. CLSI Micro Free. Available at: https://em100.edaptivedocs.net/Login.aspx. Accessed 7 April 2026.

21. Breakpoint Implementation Toolkit (BIT) | Resources | CLSI. Available at: https://clsi.org/resources/breakpoint-implementation-toolkit/. Accessed 7 April 2026.

22. Acute Care / Critical Access Hospitals (ACH) | NHSN | CDC. 2026. Available at: https://www.cdc.gov/nhsn/acute-care-hospital/index.html. Accessed 7 April 2026.

23. Patel RH, Goldin J. Medical Error Prevention and Root Cause Analysis. In: StatPearls. Treasure Island (FL): StatPearls Publishing, 2026. Available at: http://www.ncbi.nlm.nih.gov/books/NBK570638/. Accessed 7 April 2026.

24. Livorsi DJ, Drainoni M-L, Reisinger HS, et al. Leveraging implementation science to advance antibiotic stewardship practice and research. Infect Control Hosp Epidemiol **2022**; 43:139–146.

25. SHEA. SHEA. Available at: https://shea-online.org/guidance/research-methods-in-healthcare-epidemiology-and-antimicrobial-stewardship-mathematical-modeling/. Accessed 7 April 2026.
